# Impact of depressed skull fracture surgery on outcome of head injury patients

**DOI:** 10.12669/pjms.341.13184

**Published:** 2018

**Authors:** Shakeel Ahmad, Ali Afzal, Lal Rehman, Farrukh Javed

**Affiliations:** 1 Dr. Shakeel Ahmad, MBBS. Department of Neurosurgery, Jinnah Postgraduate Medical Center, Karachi, Pakistan; 2 Dr. Ali Afzal, MBBS. Department of Neurosurgery, Jinnah Postgraduate Medical Center, Karachi, Pakistan; 3 Dr. Lal Rehman, FCPS. Department of Neurosurgery, Jinnah Postgraduate Medical Center, Karachi, Pakistan; 4 Dr. Farrukh Javed, MBBS. Department of Neurosurgery, Jinnah Postgraduate Medical Center, Karachi, Pakistan

**Keywords:** Depressed skull fracture, Head injury, Glasgow Outcome Scale

## Abstract

**Objective:**

To assess outcomes in surgically managed patients with depressed skull fractures and associated moderate to severe head injury.

**Methods:**

The study was conducted in the Department of Neurosurgery Jinnah Postgraduate Medical Centre, Karachi, from January 2016 to December 2017. We analyzed 90 patients with depressed skull fracture managed surgically from January 2015 to December 2016. The patients selected for this study belonged to all age groups with clinically palpable depressed skull fracture confirmed by CT brain with bone window. Outcome was assessed by Glasgow outcome score.

**Results:**

Total 90 patients were included in the study. Sixty (66.7%) were male and 30 (33.3%) were female with mean age of years 27.58+11.329. Among 90 patients, 38.8% were aged between 21 and 30 years. Road traffic accident was seen in 72 (80%) patients. The commonest site of fracture was frontal region in 50 patients (55.6%). GCS improved post operatively on comparison to preoperative. Five patients expired.

**Conclusion:**

Depressed skull fracture is common neuro surgical issue. Timely surgical management gives excellent results by decreasing morbidity and mortality.

## INTRODUCTION

Incidence of trauma increased both in developing and developed countries especially in congested cities because of high traffic flow. This makes it a worldwide health and social issue.^1^ Traumatic head injury is a serious issue around the world.^2^,^3^ In majority of head injury cases, the incidence of depressed skull fracture is increasing, requiring trained staff and modern equipments for better care to save the lives of the patients.^4^ The patients are victims of road traffic accidents, assaults or other high-energy collisions.^5^ The introduction of Advanced Trauma Life Support (ATLS) training program, helped in better understanding of management of head injury patients.^6^ Depressed skull fracture usually occurs following high-speed impact with a small object. The outer and inner tables of skull typically break concurrently.^7^ Depressed skull fracture over superior sagittal sinus (SSS) is the commonest type of dural venous sinus injury with significant morbidity and mortality. Significant dural sinus injury occurs in 1.5 to 5% of all head injury cases.^8^ Compound depressed fractures are surgical emergencies which should be treated promptly. Early definitive diagnosis and management of skull fracture decreases morbidity and mortality.^9^ The aim of this study was to develop a plan for better surgical management of depressed skull fracture.

## METHODS

The study was conducted in the Department of Neurosurgery Jinnah Postgraduate Medical Centre, Karachi, from January 2016 to December 2017, with approval of Institutional Review Board. A total of 90 patients with depressed fractures with and without associated polytrauma were selected for this study belonging to all age groups. These had moderate to severe head injury as defined by GCS <12 and also included seven case of polytrauma. Pregnant patients, patients having previous history of head injury, and patients with head injury associated with systemic disease such as diabetes and hypertension were excluded. All polytrauma patients had long bone fractures or rib fractures and were managed conservatively. All information's were recorded in proforma. In all cases, X-Ray skull and CT brain with bone window was done to confirm the site of skull fracture. All patients in the study were managed operatively. Surgical modalities include elevation of closed as well as open depressed fracture, dural repair was done for dural tear and drainage of extradural hematoma or subdural hematoma. Outcome of surgical management was assessed by Glasgow outcome score. The patients were also examined for complications (neurological deficit, CSF leak, wound infection, death). The patients were discharged after 5–15 days, depending on the severity of head injury. Follow-up of all patients was carried out for three month to study the outcome of surgical management and complications.

Data was analyzed using SPSS version 22. All the data was expressed as mean ± SD (standard deviation) and percentage (%), as appropriate. The statistical significance of differences between the values was assessed by Chi-square test and p-value of < 0.05 was considered statistically significant.

## RESULTS

Total 90 patients of depressed skull fracture were enrolled in the study. Sixty patients (66.7%) were male and 30(33.3%) were female with mean age of years 27.58±11.329. Of 90 patients, 28(31%) were aged below 20 years. The peak incidence was observed in patients belonging to 21–30 age groups. The commonest cause of depressed skull fracture was road traffic accident, seen in 72 (80%) patients; whereas in 12 (13.3%), mode of injury was fall from height and in 6 (6.7%) fall of object on patient. All patients presented with the main complaint of altered level of conscious followed by vomiting and fit as shown in Table-I.

**Table-I:** Age and sex distribution, mode of injury and mode of presentation.

	Characteristics	Frequency	Percentage	Mean ± SD
Age (years)	0-10	2	2.2	27.58±11.329
	11-20	26	28.8	
	21-30	35	38.8	
	31-40	13	14.4	
	41-50	9	10	
	51-55	5	5.8	
Sex	Male	60	66.7	
	Female	30	33.3	
Mode of Injury	Road traffic accident	72	80	
	Fall from height	12	13.3	
	Fall of object on patient	6	6.7	
Mode of presentation	Altered level of Consciousness	90	100	
	Vomiting	14	15.6	
	Fits	13	14.4	
	ENT bleeding	7	7.8	

**Fig. 1:**
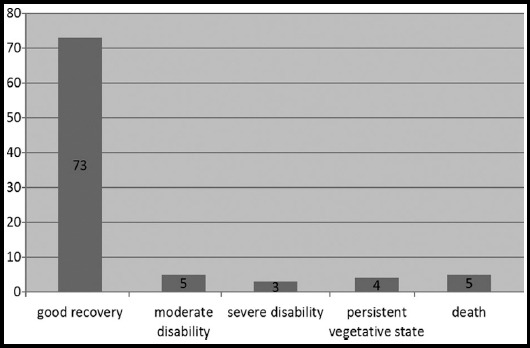
Glasgow outcome score.

The commonest site of fracture was frontal region seen in 50 cases (55.6%) as shown in Table-II. Compound fracture of skull was found in 70 cases (77.7%) whereas simple fracture was found in 20 cases (22.3%). Dura was intact in 46 cases (51.1%), but was breached in 44 cases (48.9%). Associated brain injuries like extradural hematoma was reported in ten patients (11.1%), subdural hematoma in five patients (5.6%), involvement of venous sinuses in 5 cases (5.6%) and brain contusions in 35 cases (38.8%). Preoperatively, there were 70 patients (66.7%) with moderate head injury (GCS:8-12) and 20 patients(33.3%) were with severe head injury (GCS<8). All patients underwent surgery and in all cases, antibiotics and antiepileptics were given. Postoperatively outcome was assessed by Glasgow outcome score as shown in Fig.1. Complication occurred in 16 patients, 4 patients (4.4%) had neurological deficit, four (4.4%) patients had wound infection, three patients (3.3%) had CSF leak and five patients (5.6%) expired.

**Table-II:** Sites of fracture and type of fractures distribution.

	Characteristics		Frequency	Percentage	P-value
Sites of fracture	Frontal		50	55.6	0.181
	Parietal		14	15.6	
	Temporal		11	12.2	
	Occipital		15	16.7	
Type of fractures	Open fracture	Dural tear	40	44.4	0.061
		Dural intact	30	33.3	
		Total	70	77.7	
	Closed fracture	Dural tear	4	4.4	0.998
		Dural intact	16	17.8	
		Total	20	22.2	

**Table-III:** Associated brain injuries.

	Characteristics		Frequency	Percentage	P-value
Associated Brain injuries	Intracranial hematoma				
		EDH	10	11.2	0.000
		SDH	5	5.6	
	Involvement of venous sinus		5	5.6	
	Brain contusions		35	38.8	

## DISCUSSION

Trauma is a major problem both in the developed and developing countries. Head injury is a major risk factor responsible for mortality in young population.^10^ We analyzed 90 cases of depressed skull fractures treated in context of mode of injury, mode of presentation, site of fracture, type of fracture, associated brain injuries, surgical management and outcome of patients. Various aspects were compared with similar work and studies carried out in the past.

In a depressed skull fracture, the major bone depression can occur at the interface of fracture. Majority of depressed skull fractures occur over frontoparietal region as bone is comparatively thin and prone to trauma.^11^ Surgical elevation is the standard treatment of depressed skull fractures if depression is equal or more than thickness of normal adjacent skull.^12^ Surgical elevation of depressed skull fractures over a venous sinus may cause fatal hemorrhage if a depressed fragment has been plugging a sinus tear.^13^ The surgical treatment for depressed skull fractures located over major venous sinuses is controversial. However surgical decompression is indicated if sinus obliteration and related intracranial hypertension are present clinically and radiologically.^14^,^15^ Early surgical management on a compound depressed skull fracture over the superior sagittal sinus with epidural hematoma may give a better outcome.^16^

As regards the mode of trauma, assault was the commonest cause found by Hossain et al.^17^ and AI-Haddad and Kirollos^10^ whereas in the study by Ali et al.^1^ fall from height was the main cause of injury (51.94%). In Rolekar et al.^6^ study, road traffic accident was the main cause of injury (60%). Ozer FD et al. reported road traffic accident as the commonest cause of depressed skull fracture followed by assault.^18^ In a study by Braakman^19^ fifty one percent of the patients had road traffic accident followed by fall from height in 26% patients. In our study, road traffic accident was the main cause of injury (80%) that is most probably because of not following traffic rules consistently.

As for as the presentation of depressed skull fracture is concerned, it presents with loss of consciousness, fits, vomiting and ENT bleed. In Rolekar et al.^6^ study, the most frequent cause of presentation was unconsciousness (56%) followed by ENT bleeding (34%), whereas in Ali et al.^1^ and Hossain et al.^17^ studies it was unconsciousness in 40% and 25% cases, respectively. In our study, the most common mode of presentation was altered level of consciousness (100%) followed by vomiting (15.6%), fits (14.4%) and ENT bleeding (7.8%).

Skull fractures can occur at any site of skull depending upon the mode they are caused. In Rolekaretal^6^ study the frontal region was the most common site of injury in 52% patients. In Ali's et al. Study^1^, parietal was the most common site of injury followed by temporal region. In studies by Braakman^19^ and Mehdi et al.^20^, frontal region was the commonest site of fracture in accident. In our study frontal region was the most common site of injury in 55.6% patients followed by occipital in 16.7%, parietal in 15.6% and temporal in 12.2% cases.

In Rolekar et al.^6^. study 68% of the fractures were compound fractures. However, in studies by Ali et al.^1^ Braakman^19^, Hossain et al.^17^ compound fractures were found in 73.5%, 86%, and 43.64% patients, respectively. In our study, 77.7% were open fractures while 22.2% of the fractures were closed fractures.

In depressed skull fractures, dura is breached in considerable number of cases as has been reported by different studies. In Hossain et al. study^17^ of 93 patients, 67 patients were operated with 17 patients (25%) having dural tears. In Braakman^19^ study of 225 patients, 44% had dura tear. In our study, 48.9% patients had dural tear which is comparable to Braakman study.^19^

Most often depressed skull fractures are associated with underlying hematoma, contusion and venous sinus involvement which might be much dangerous for patients as compared to fracture alone. In Mehdi et al. study^20^, 31 patients had brain contusion and four had SDH. In Hossain et al. study^17^, 22% patients had EDH and 31% had brain contusion. In Ali et al.study^1^, 10.78% patients had ICH and 3.9% had involvement of venous sinus. In our study, 35(38.8%) patients had brain contusions; 10(11.2%) patients had extradural hematoma and five(5.6%) patients had subdural hematoma and five(5.6%) patients had involvement of venous sinuses.

Prompt diagnosis and management of skull fracture reduces morbidity and mortality as well as help in restoring maximal functional and aesthetic rehabilitation. We saw good recovery in 73(81.1%) patients and moderate disability in five(5.5%) patients similar to findings of Rolekar^6^ and AI-Haddad and Kirollos^10^ which shows intervention in even in moderate to severe head injury can be beneficial. Surgical interventions are done to benefit the patients, but complications can also occur as reported by different studies. In Rolekar et al. study^6^ postoperative infection was found in 16.66% cases, 8% patients had neurological deficit like hemiparesis whereas in the study by AI-Haddad and Kirollos^10^ 12.3% patient had episodes of postoperative epilepsy. In our study complications occurred in sixteen patients, four patients (4.4%) had neurological deficit, four patients (4.4%) had wound infection, three patients (3.3%) had CSF leak and five patients (5.6%) expired.

## CONCLUSION

Patients with moderate to severe head injury can be fatal if not managed timely. Depressed fracture if not managed, may affect the outcome as well in these patients. Surgery for depressed fracture should be done because early diagnosis and management of depressed skull fracture decreases morbidity and mortality.

### Authors` Contribution

**SA** conceived and designed the study.

**AA** did statistical analysis & editing of manuscript.

**LR** did review and final approval of manuscript.

**FJ** did data collection and manuscript writing.
